# 

**Published:** 1859-01

**Authors:** 


No. VIII. JANUARY—1859. Vol. XIV.

### BUFFALO MEDICAL JOURNAL

AND

### Monthly Review

OF

### MEDICAL AND SURGICAL SCIENCE.

AUSTIN FLINT, Jr., M. D.,

EDITOR.

BUFFALO, N. Y.

A. I. MATHEWS, PUBLISHER,

No. 220 Main Street.

Price $2.50, payable in advance, or $3.00 if not paid till the end of the year.

